# Bariatric Surgery Utilization Trends in the United States Following COVID-19 and the 2022 ASMBS/IFSO Guideline Expansion: An Interrupted Time Series Analysis

**DOI:** 10.3390/jcm15124591

**Published:** 2026-06-13

**Authors:** Abdulrahman A. Alsuhibani

**Affiliations:** Department of Pharmacy Practice, College of Pharmacy, Qassim University, Buraydah 51452, Qassim, Saudi Arabia; aa.alsuhibani@qu.edu.sa

**Keywords:** obesity, bariatric surgery, sleeve gastrectomy, COVID-19, GLP-1, surgical utilization trends, interrupted time series

## Abstract

**Background:** In the US, obesity is still a serious public health issue. Bariatric surgery utilization has recently been influenced by the COVID-19 pandemic and the 2022 American Society for Metabolic and Bariatric Surgery (ASMBS)/International Federation for the Surgery of Obesity and Metabolic Disorders (IFSO) guideline expansion. The combined effects of these events on national utilization trends remain unclear. **Methods:** We conducted a retrospective longitudinal study using electronic medical record data from the TriNetX network, including adults aged ≥18 years from 2018 through 2024. Primary bariatric procedures were identified using validated CPT and ICD codes. Quarterly surgery rates per 100,000 adults were calculated using the number of unique adults with at least one healthcare encounter per quarter as the denominator. Level and slope changes related to the start of COVID-19 (Q2 2020) and the application of the 2022 ASMBS guidelines (Q1 2022) were evaluated using interrupted time series models. Procedure-type distributions were also evaluated. **Results:** A total of 215,072 procedures were identified. Utilization was stable before Q2 2020, when a significant decline occurred following the onset of COVID-19. Rates recovered through 2021. After Q1 2022, a modest immediate increase was observed, followed by a sustained downward trend through 2024. Sleeve gastrectomy accounted for approximately two-thirds of procedures throughout the study period. **Conclusions:** Bariatric surgery utilization was markedly disrupted by COVID-19 and showed limited sustained growth after guideline expansion. These patterns might be a reflection of more general changes in the management of obesity, such as the growing accessibility of medication.

## 1. Introduction

Obesity is a long-term, gradually worsening condition whose prevalence continues to increase worldwide. In the United States, more than 40% of adults are classified as obese, substantially increasing the risk of type 2 diabetes, cardiovascular disease, several malignancies, and premature mortality [1,2]. Although lifestyle changes continue to serve as the primary approach to treatment, weight maintenance is often difficult to achieve through behavioral strategies alone [3].

Pharmacologic management of obesity has evolved rapidly in recent years. Glucagon-like peptide-1 receptor agonists (GLP-1 RAs), such as semaglutide, and dual glucose-dependent insulinotropic polypeptide (GIP)/GLP-1 agonists, such as tirzepatide, have demonstrated significant weight reduction in randomized clinical trials [4]. Despite these advances metabolic and bariatric surgery (MBS) continues to be the most effective long-term intervention for severe obesity, consistently leading to sustained weight reduction and improvement or remission of obesity-related comorbid conditions, and reduced all-cause mortality [5,6].

The landscape of bariatric surgery utilization has undergone substantial shifts over the past several years. First, the COVID-19 pandemic led to widespread suspension of elective procedures, including MBS, resulting in abrupt reductions in surgical volume during 2020 and gradual recovery thereafter [7]. Second, in 2022, the American Society for Metabolic and Bariatric Surgery (ASMBS) and the International Federation for the Surgery of Obesity and Metabolic Disorders (IFSO) revised the eligibility guidelines for metabolic and bariatric surgery, lowering BMI thresholds and broadening surgical candidacy [8]. These changes substantially expanded the pool of potentially eligible patients and were expected to increase surgical access at the population level.

At the same time, the rapid uptake of highly effective anti-obesity pharmacotherapies has introduced a new dynamic into obesity management. Randomized clinical trials have demonstrated substantial weight reductions with glucagon-like peptide-1 receptor agonists and dual GIP/GLP-1 agonists, with outcomes approaching those historically observed with surgery in selected populations [4,9]. National prescribing data indicate a sharp increase in GLP-1 receptor agonist utilization beginning in 2021, reflecting growing clinical adoption [10]. The expanding availability of these agents may influence referral patterns, patient preferences, and healthcare resource allocation, potentially altering trends in surgical utilization, although the magnitude of this effect remains uncertain.

Despite these concurrent developments, comprehensive evaluations of bariatric surgery utilization that integrate pandemic disruption, guideline expansion, and the emergence of pharmacotherapy remain limited. Prior reports have primarily described annual aggregate volumes or short-term pandemic-related declines without examining longer-term quarterly trends or the interaction of multiple structural shifts [7,11,12]. Understanding these temporal patterns is essential for healthcare planning, surgical workforce allocation, and equitable access to obesity treatment.

## 2. Methods

### 2.1. Study Design and Population

This retrospective observational cohort study utilizes electronic medical record (EMR) data from the TriNetX research network. The study population includes adult patients (≥18 years) who underwent primary bariatric surgery procedures between 1 January 2018 and 31 December 2024. Patients undergoing laparoscopic or open bariatric surgeries were identified using Current Procedural Terminology (CPT-4), International Classification of Diseases, Ninth Revision, Clinical Modification (ICD-9-CM), and Tenth Revision, Clinical Modification (ICD-10-CM) codes (Table S2) [13,14]. Revisional bariatric operations were specifically left out (Table S3) [13,14].

### 2.2. Data Source

Patient-level data were obtained from the TriNetX platform (Cambridge, MA, USA). TriNetX is a federated health research network comprising over 84 million de-identified patient EMRs from 69 healthcare organizations (HCOs) across the United States, including academic medical centers, hospitals, primary care, and specialty treatment providers. All patient and provider data are aggregated and de-identified within the TriNetX network and comply with the HIPAA Safe Harbor de-identification standard [13,15,16,17]. Due to the de-identified nature of this data, Institutional Review Board (IRB) approval was waived.

### 2.3. Exposure

Exposure was defined as undergoing a primary MBS procedure. Eligible procedures included open or laparoscopic Roux-en-Y gastric bypass (RYGB), sleeve gastrectomy (SG), adjustable gastric banding (AGB), biliopancreatic diversion with duodenal switch (BPD/DS), vertical-banded gastroplasty (VBG), and single-anastomosis duodenal-ileal bypass with sleeve gastrectomy (SADI-S). Procedures were identified from electronic medical records using validated CPT-4, ICD-9-CM, and ICD-10-CM codes in either inpatient or outpatient settings, revisional procedures were excluded (Supplementary Materials Tables S2 and S3).

### 2.4. Outcomes

The quarterly rate of primary bariatric surgery per 100,000 adults was the main result. Rates were calculated as the number of primary procedures performed in a given quarter divided by the number of unique adults (≥18 years) with at least one healthcare encounter during the same quarter, multiplied by 100,000. Secondary outcomes included the distribution of procedure types (SG, RYGB, Others) and subgroup-specific utilization patterns by demographic and clinical characteristics.

### 2.5. Statistical Analysis

Baseline patient demographics and clinical characteristics were summarized as means with standard deviations (SD) for continuous variables and as frequencies with percentages for categorical variables. Comparisons across study periods—pre-COVID (2018 Q1–2020 Q1), COVID (2020 Q2–2021 Q4), and post-guideline expansion (2022 Q1–2024 Q4)—were conducted using chi-square tests for categorical variables and one-way ANOVA for continuous variables. Post hoc pairwise *t*-tests with multiple-testing correction were applied where appropriate.

Interrupted time series (ITS) regression was used to evaluate changes in quarterly bariatric surgery rates at two predefined interruption points: the onset of the COVID-19 pandemic (Q2 2020) and implementation of the 2022 ASMBS/IFSO guideline expansion (Q1 2022). Time was modeled as sequential calendar quarters beginning in Q1 2018. The model was specified as:

Y_t_ = β_0_ + β_1_(Time)_t_ + β_2_(COVID_Level)_t_ + β_3_(Time_After_COVID)_t_ + β_4_(Guideline_Level)_t_ + β_5_(Time_After_Guideline)_t_ + ε_t_

where Y_t_ is the quarterly bariatric surgery rate per 100,000 adults; Time is the sequential quarter index; COVID_Level and Guideline_Level are indicator variables coded 0 before and 1 from Q2 2020 and Q1 2022, respectively; and Time_After_COVID and Time_After_Guideline denote the number of quarters elapsed since each interruption. β_1_ estimates the baseline (pre-COVID) trend, β_2_ and β_4_ the immediate level changes, and β_3_ and β_5_ the post-interruption slope changes. To provide valid inference in the presence of serial correlation and heteroskedasticity, the models were estimated with Newey–West heteroskedasticity- and autocorrelation-consistent (HAC) standard errors using four quarterly lags. As a sensitivity analysis, seasonality was modeled with quarter-of-year indicator variables (Q2–Q4; Q1 as the reference category); the seasonally adjusted estimates were consistent with the primary model (Table 1).

Subgroup analyses examined trends by procedure type (SG, RYGB, others) and explored utilization patterns by race/ethnicity, insurance status, and geographic region.

All analyses were conducted using R version 4.3.1 (R Foundation for Statistical Computing, Vienna, Austria). Figures were generated using the ggplot2 package in R and Microsoft Excel (Microsoft Corporation, Redmond, WA, USA). A two-sided *p*-value < 0.05 was considered statistically significant.

### 2.6. Ethical Considerations

TriNetX provides fully de-identified data in compliance with HIPAA’s Safe Harbor standard. As this study used aggregated and anonymized data, review by an institutional review board (IRB) was not required.

## 3. Results

### 3.1. Study Population

Between 2018 and 2024, a total of 215,072 adult patients underwent primary bariatric surgical procedures across participating healthcare organizations (HCOs) in the TriNetX network. Quarterly procedure volumes ranged from approximately 4600 to 8700 cases. Throughout the study period, the analytic denominator, adults aged ≥18 years with at least one healthcare encounter per quarter, remained relatively stable at approximately 16–18 million individuals. This data enabled calculation of standardized quarterly utilization rates per 100,000 adults, which served as the primary outcome for interrupted time series analyses.

Baseline patient demographics and comorbidities are summarized in Table 2, with comparisons across the pre-COVID (2018 Q1–2020 Q1), COVID (2020 Q2–2021 Q4), and post-guideline (2022 Q1–2024 Q4) periods. Overall, patients were predominantly female (~70%). Mean age declined from 55.6 years in the pre-COVID period to 50.9 years in the post-guideline period. Most patients were White (61%), followed by Black (21%) and Hispanic (13%) individuals. Regionally, the South (≈33%) and Northeast (≈29%) accounted for the largest proportions. Additional descriptive statistics are provided in Supplementary Table S1.

### 3.2. Comparative Analyses

Modest but statistically significant differences were found when comparing study periods in demographic shifts (Table 2). Mean age declined over time (ANOVA *p* < 0.001). The proportion of female patients increased slightly, while male and unknown sex categories remained stable (overall *p* < 0.001). The proportion of White patients decreased from 65.3% pre-COVID to 60.9% post-guideline, whereas Black and Hispanic patient proportions increased (overall *p* < 0.001). Regional representation remained largely consistent, although small shifts reached statistical significance, likely reflecting the large sample size.

**Figure 1:**
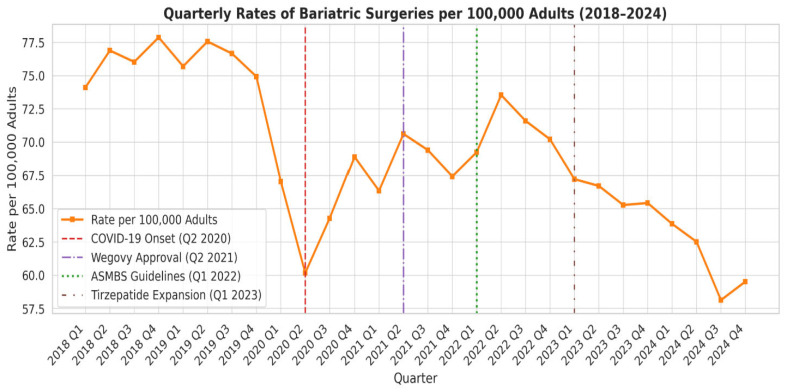
Quarterly rates of primary bariatric surgery performed across participating health care organizations within the TriNetX network from Q1 2018 through Q4 2024.

Quarterly rates of primary bariatric surgery per 100,000 adults are presented in Figure 1. Rates closely paralleled raw procedure counts, with a baseline average of approximately 78 per 100,000 adults in early 2018. A marked decline occurred in Q2 2020, coinciding with the onset of the COVID-19 pandemic, followed by a recovery through 2021. After the release of the 2022 ASMBS guidelines in Q1 2022, rates increased briefly before entering a sustained downward trend through the end of the study period.

**Figure 2:**
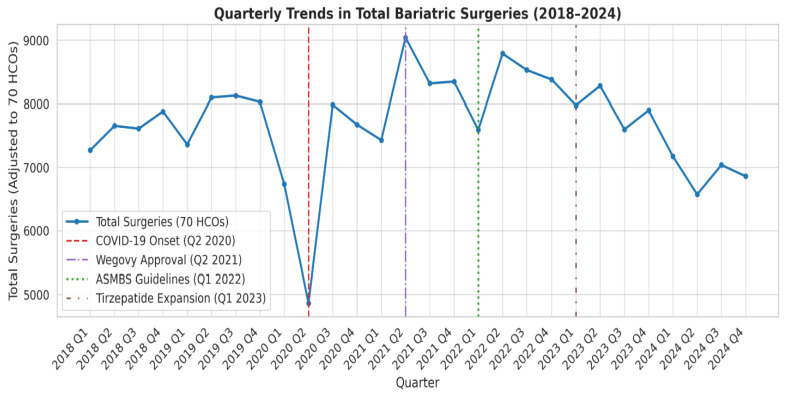
Quarterly Total Number of primary bariatric surgery performed across participating health care organizations within the TriNetX network from Q1 2018 through Q4 2024.

Quarterly total procedure counts are shown in Figure 2. Volumes were stable during 2018–2019, declined sharply in Q2 2020, and rebounded in subsequent quarters. After peaking in 2021 and early 2022, procedure volumes declined steadily through 2023 and 2024.

Table 1 presents interrupted time series regression results. Prior to Q2 2020, no significant baseline slope was observed (β −0.55; 95% CI −1.26 to 0.16; *p* = 0.128). At the onset of COVID-19, a large immediate level decline of −11.07 per 100,000 adults was observed (95% CI −14.84 to −7.31; *p* < 0.001), followed by a significant positive slope change indicating recovery (+1.76 per 100,000 per quarter; 95% CI 0.53 to 2.98; *p* = 0.005).

**Figure 3:**
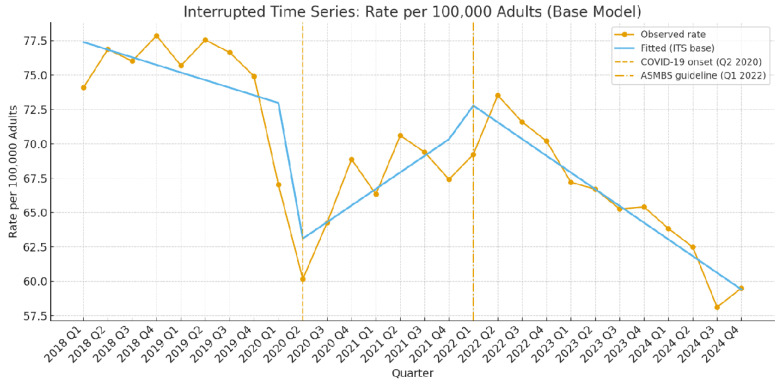
Interrupted Time Series (ITS) analysis of quarterly bariatric surgery rates per 100,000 adults: Base model (2018–2024).

**Figure 4:**
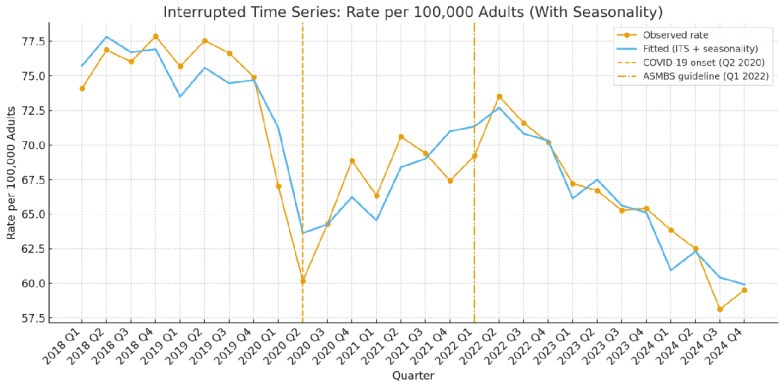
Interrupted Time Series (ITS) analysis of quarterly bariatric surgery rates per 100,000 adults: Seasonally adjusted model (2018–2024).

Following implementation of the 2022 ASMBS guidelines in Q1 2022, there was a modest immediate level increase (+3.66 per 100,000 adults; 95% CI 1.17 to 6.16; *p* = 0.004), followed by a significant negative slope change (−2.42 per 100,000 per quarter; 95% CI −3.26 to −1.58; *p* < 0.001). Seasonally adjusted models yielded nearly identical estimates, supporting robustness (Table 1). Model fits are illustrated in Figure 3 and Figure 4.

### 3.3. Procedure-Type Analysis

**Figure 5:**
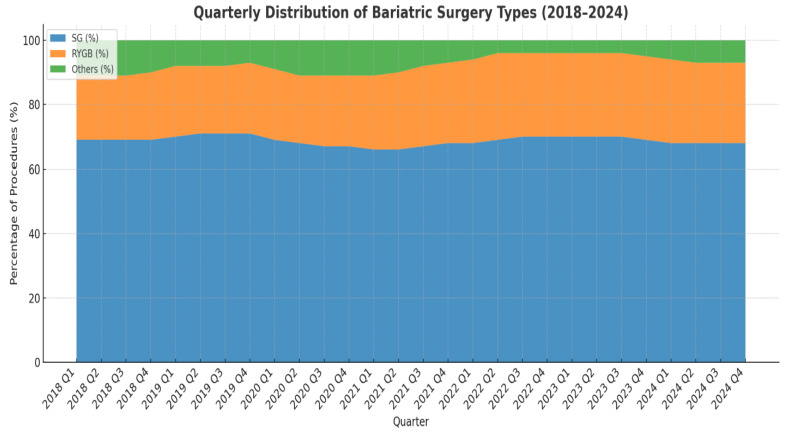
Quarterly Distribution of Primary Bariatric Surgery Types, 2018–2024.

The distribution of bariatric procedure types is shown in Figure 5, with detailed quarterly proportions provided in Supplementary Figure S1. Sleeve gastrectomy consistently accounted for approximately 65–70% of procedures, Roux-en-Y gastric bypass for approximately 25%, and all other procedures for less than 10%. These proportions remained relatively stable over time, suggesting that sleeve gastrectomy volumes were the main driver of overall usage trends.

## 4. Discussion

This study provides a comprehensive evaluation of bariatric surgery utilization in the United States from 2018 through 2024, spanning the pre-pandemic period, the COVID-19 disruption, and the post-2022 ASMBS/IFSO guideline era. Using a large real-world EMR network and interrupted time series analysis, we identified a marked pandemic-related disruption, a modest short-term increase following guideline expansion, and a subsequent sustained decline in surgical rates.

Consistent with national ASMBS estimates, bariatric surgery volumes demonstrated stable growth prior to 2020 [7,18]. The onset of the COVID-19 pandemic abruptly altered this trajectory, as elective procedures were widely suspended across health systems. A substantial reduction in bariatric and metabolic surgery activity during 2020, with gradual recovery, thereafter, has been documented in national estimates and international guidance statements [7,11]. These conclusions are supported by our interrupted time series results, demonstrating both an immediate level decrease and a subsequent positive slope change consistent with system recovery.

The 2022 ASMBS/IFSO guidelines expanded eligibility criteria by lowering BMI thresholds and broadening indications for surgery [8]. Although such changes were anticipated to increase utilization, our analysis identified only a modest immediate rise in surgical rates that was not sustained over subsequent quarters. Limited persistence of this effect may reflect heterogeneity in institutional adoption as well as constraints related to coverage policies and referral pathways [8,16].

An additional contextual factor is the expanding availability of highly effective anti-obesity pharmacotherapies. Randomized clinical trials have demonstrated substantial weight reduction with semaglutide and tirzepatide, with outcomes approaching those historically observed with surgery in selected populations [4,9]. At the population level, prescribing data show rapid growth in GLP-1 receptor agonist use beginning in 2021.10 While causal inference cannot be established in this observational analysis, the emergence of highly effective anti-obesity pharmacotherapies represents an important concurrent development in obesity management during the study period. Because patient-level medication data were not available, the potential contribution of pharmacotherapy to the observed surgical trends cannot be directly evaluated and should be regarded as hypothesis-generating. Notably, the incomplete rebound in surgical utilization during 2021–2022 preceded the period of widest pharmacotherapy adoption and is therefore more plausibly attributable to persistent pandemic-related disruption, procedural backlogs, staffing constraints, and coverage and referral barriers, whereas the continued decline through 2023–2024 coincided with broader uptake of these agents. Bariatric surgeons increasingly participate in multidisciplinary obesity care and may initiate or recommend pharmacotherapy; however, the data do not capture prescriber specialty, precluding assessment of how medical and surgical management are integrated at the clinician level.

Procedure-specific analyses demonstrated that sleeve gastrectomy remained the predominant operation throughout the study period, accounting for approximately two-thirds of procedures, while Roux-en-Y gastric bypass comprised roughly one-quarter. This distribution aligns with national reporting and reflects longer-term shifts in procedure mix [7,17].

This study has several strengths. It leverages a large multicenter EMR network and captures more than six years of quarterly data across major healthcare disruptions, while applying interrupted time series methodology to quantify both immediate and slope-level changes. Several limitations should also be considered. As with any multi-institutional EMR network, coding practices and data capture may vary across contributing health systems. Quarterly rates were calculated using adults with at least one healthcare encounter as the denominator; reduced in-person visits and the rapid expansion of telehealth during the COVID-19 pandemic may have influenced rate estimates, although the denominator remained relatively stable (~16–18 million adults per quarter) throughout the study period, suggesting any such effect was modest; to the extent it contracted during the acute pandemic phase, this would have biased the calculated rate upward and thus understated, rather than exaggerated, the observed decline. Patient-level pharmacotherapy data were also unavailable, so we could not determine whether patients had received GLP-1 receptor agonists or other anti-obesity medications before surgery, the duration of medical therapy preceding surgical referral, or the proportion of patients in whom medical management was unsuccessful; nor could we adjust for additional patient-level drivers such as insurance coverage and socioeconomic status [18,19]. Studies incorporating patient-level medication histories and treatment sequencing represent an important direction for future research. Finally, the observational design precludes causal inference, and findings should be interpreted as temporal associations.

Overall, these findings underscore the profound impact of COVID-19 on bariatric surgery delivery, the limited sustained effect of guideline expansion on utilization, and the growing influence of pharmacotherapy in obesity care. Ongoing surveillance will be essential to understand how surgical and medical treatments are integrated as obesity management continues to evolve.

## 5. Conclusions

Bariatric surgery utilization in the United States from 2018 to 2024 was substantially influenced by the COVID-19 pandemic and the 2022 ASMBS/IFSO guideline update. A marked decline occurred at the onset of COVID-19, followed by recovery during 2021. Although guideline expansion was associated with a modest immediate increase in utilization, this effect was not sustained, and rates declined through 2024. Sleeve gastrectomy remained the dominant procedure throughout the study period. The limited long-term growth observed after guideline expansion likely reflects multiple factors, including ongoing healthcare-system recovery, barriers to surgical access, evolving referral patterns, and the broadening range of obesity treatment options. Continued monitoring will be important to understand how medical and surgical therapies are integrated within obesity care.

## Figures and Tables

**Table 1:** Interrupted time series regression estimates for quarterly bariatric surgery rates (2018–2024).

Model Term	Base Model β (95% CI)	*p*-Value	Seasonal Model β (95% CI)	*p*-Value
Intercept	77.95 (74.47, 81.43)	<0.001	76.30 (73.65, 78.94)	<0.001
Baseline slope (time)	−0.55 (−1.26, 0.16)	0.128	−0.56 (−1.14, 0.02)	0.060
COVID-19 level change (Q2 2020)	−11.07 (−14.84, −7.31)	<0.001	−11.46 (−15.02, −7.90)	<0.001
COVID-19 slope change	+1.76 (0.53, 2.98)	0.005	+1.75 (0.54, 2.96)	0.005
Guideline level change (Q1 2022)	+3.66 (1.17, 6.16)	0.004	+4.53 (2.08, 6.98)	<0.001
Guideline slope change	−2.42 (−3.26, −1.58)	<0.001	−2.49 (−3.42, −1.56)	<0.001

β coefficients represent absolute changes in quarterly bariatric surgery rates per 100,000 adults. The intercept reflects the estimated baseline rate at the start of the study period (Q1 2018). Baseline slope represents the pre-COVID quarterly trend. Level changes represent immediate shifts in rates at the time of interruption. Slope changes represent changes in quarterly trends following interruption. Seasonal model includes quarter-of-year indicator variables. Newey–West HAC standard errors with four quarterly lags were used. *p*-values < 0.05 are considered statistically significant.

**Table 2:** Baseline demographic and clinical characteristics of adult patients undergoing primary bariatric surgery, stratified by study period (2018–2024).

Characteristic	Pre-COVID	COVID	Post-Guideline	Δ (Post vs. Pre)	*p*-Value
**Age, mean (years)**	55.6	53.1	50.9	−4.7 yrs	<0.001
**Sex (%):**					
**Female**	68.3	69.0	70.2	+1.9	<0.001
**Male**	29.1	27.8	28.0	−1.1	
**Unknown sex**	2.6	3.2	1.8	−0.8	
**Race (%):**					
**White**	65.3	61.7	60.9	−4.4	<0.001
**Black**	18.6	20.1	20.9	+2.3	
**Other**	16.2	18.2	18.2	+2.0	
**Ethnicity (%):**					
**Hispanic**	10.3	10.5	13.0	+2.7	<0.001
**Non-Hispanic**	69.7	69.5	67.2	−2.5	
**Unknown**	19.9	20.0	19.8	−0.1	
**Region (%):**					
**Northeast**	30.6	28.7	29.1	−1.5	<0.001
**Midwest**	16.8	19.7	20.1	+3.3	
**South**	33.0	33.8	32.8	−0.2	
**West**	12.0	11.2	11.6	−0.4	
**Unknown**	4.7	3.1	3.6	−1.1	
**Ex-US**	3.0	2.8	3.0	0.0	

Pre-COVID period defined as 2018 Q1–2020 Q1; COVID period as 2020 Q2–2021 Q4; Post-Guideline period as 2022 Q1–2024 Q4. Continuous variables are presented as mean ± standard deviation (SD); categorical variables as number (percentage). Δ represents absolute difference between Pre-COVID and Post-Guideline values (percentage points for categorical variables and mean years for age). *p*-values derived from one-way ANOVA for continuous variables and chi-square tests for categorical variables. Due to the large sample size, small differences may reach statistical significance; effect size and clinical relevance should be considered in interpretation.

## Data Availability

The data presented in this study are available on request from the corresponding author.

## Supplementary Materials

The following supporting information can be downloaded at: https://www.mdpi.com/article/10.3390/jcm15124591/s1, Table S1: Additional Descriptive Statistics for Baseline Demographic and Clinical Characteristics of Adult Patients Undergoing Primary Bariatric Surgery, Stratified by Study Period (2018–2024); Table S2: Diagnosis and Procedure Codes for Bariatric Surgery and Obesity; Table S3: Procedure Codes Used to Identify Open and Laparoscopic Revisional Bariatric Surgery; Figure S1: Quarterly Distribution of Primary Bariatric Surgery Types in the United States, 2018–2024.
