# Supplementary material for: Bariatric Surgery Utilization Trends in the United States Following COVID-19 and the 2022 ASMBS/IFSO Guideline Expansion: An Interrupted Time Series Analysis
Source: J Clin Med. 2026 Jun 13;15(12):4591. doi: 10.3390/jcm15124591 (PMC13302672; doi:10.3390/jcm15124591)
Supplement: Supplementary file 1 [file jcm-15-04591-s001.zip › jcm-4282934-supplementary.pdf]

Table S1. Additional Descriptive Statistics for Baseline Demographic and Clinical Characteristics of Adult Patients Undergoing Primary Bariatric Surgery, Stratified by Study Period (2018–2024)

Quarter	Average Age	Sex - Female (%)	Sex - Male (%)	Sex - Unknown (%)	Race - White (%)	Race - Black (%)	Race - Other (%)	Ethnicity - Not Hispanic or Latino (%)	Ethnicity - Hispanic (%)	Ethnicity - Unknown (%)	Region - Northeast (%)	Region - Midwest (%)	Region - South (%)	Region - West (%)	Region - Unknown (%)	Region - Ex-US (%)
**2018 Q1**	56	69	29	2	68	18	14	70	11	19	31	16	32	12	6	3
**2018 Q2**	56	68	29	3	65	19	16	70	10	20	31	16	32	12	6	3
**2018 Q3**	56	68	29	3	65	18	17	70	10	20	31	17	32	12	5	3
**2018 Q4**	56	68	29	3	65	18	17	70	10	20	31	16	33	12	5	3
**2019 Q1**	56	69	30	1	66	19	15	70	11	19	30	17	34	12	4	3
**2019 Q2**	55	69	29	2	65	19	16	69	10	21	31	16	34	12	4	3
**2019 Q3**	55	68	29	3	64	19	17	69	10	21	31	17	33	12	4	3
**2019 Q4**	55	69	28	3	65	19	16	69	10	21	30	18	33	12	4	3
**2020 Q1**	55	67	30	3	65	18	17	71	11	18	29	18	34	12	4	3
**2020 Q2**	56	64	33	3	64	18	18	70	10	20	29	19	34	12	3	3
**2020 Q3**	54	68	28	4	62	20	18	71	9	20	29	20	34	11	3	3
**2020 Q4**	53	68	28	4	62	20	18	69	10	21	28	20	34	11	4	3
**2021 Q1**	53	70	27	3	64	20	16	71	10	19	27	21	35	11	3	3
**2021 Q2**	52	70	26	4	61	20	19	68	11	21	28	20	34	12	3	3
**2021 Q3**	52	70	27	3	60	21	19	69	11	20	30	19	34	11	3	3

**2021 Q4**	52	71	28	1	60	21	19	69	12	19	30	19	34	11	3	3
**2022 Q1**	52	71	28	1	61	22	17	70	13	17	31	18	33	12	3	3
**2022 Q2**	52	70	27	3	61	21	18	69	11	20	30	18	34	12	3	3
**2022 Q3**	51	71	27	2	60	21	19	68	12	20	29	18	35	11	4	3
**2022 Q4**	51	72	26	2	60	21	19	67	13	20	28	19	36	10	4	3
**2023 Q1**	51	71	28	1	62	21	17	67	14	19	28	19	36	9	5	3
**2023 Q2**	50	70	28	2	60	22	18	66	14	20	27	20	34	11	5	3
**2023 Q3**	50	70	27	3	59	22	19	66	13	21	28	20	33	12	4	3
**2023 Q4**	50	69	28	3	60	21	19	67	13	20	30	20	32	11	4	3
**2024 Q1**	51	69	28	3	60	22	18	67	13	20	30	21	31	13	2	3
**2024 Q2**	51	70	29	1	61	20	19	66	14	20	31	22	29	13	2	3
**2024 Q3**	51	70	30	0	64	18	18	66	14	20	29	23	28	14	3	3
**2024 Q4**	51	69	31	0	64	19	17	67	13	20	28	25	27	14	3	3

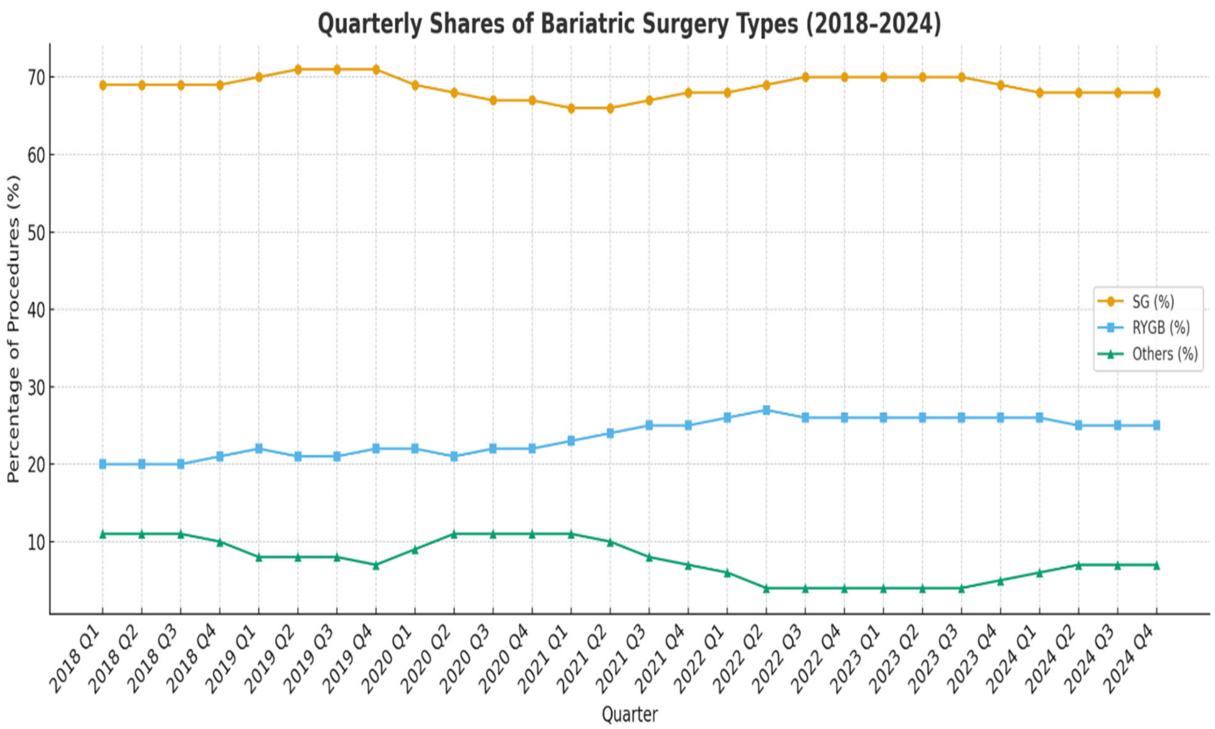

Figure S1. Quarterly Distribution of Primary Bariatric Surgery Types in the United States, 2018–2024.

Table S2. Diagnosis and Procedure Codes for Bariatric Surgery and Obesity.

Variable	Codes
**Open Roux-en-Y gastric bypass (RYGB)**	CPT-4: 43846, 43847<br>ICD-9-CM: 44.31, 44.39<br>ICD-10: 0D16078,
**Laparoscopic Roux-en-Y gastric bypass (RYGB)**	CPT-4: 43644, 43645<br>ICD-9-CM: 44.38<br>ICD-10: 0D16479, 0D1647A, 0D164J9, 0D164JA, 0D164K9, 0D164KA, 0D164Z9,<br>0D164ZA, 0D164ZB
**Open Sleeve gastrectomy (SG)**	CPT: 43843,<br>ICD-9-CM: 43.89, 44.69<br>ICD-10: 0DQ60ZZ ‹0DB64Z3, 0DV64CZ, 0DB63Z3, 0DB60Z3
**Laparoscopic Sleeve gastrectomy (SG)**	CPT: 43775<br>ICD-9-CM: 43.82<br>ICD-10: 0DB64Z3
**Laparoscopic adjustable gastric band (AGB)**	CPT-4: 43770, S2082<br>ICD-9: 44.95<br>ICD-10: 0DV64CZ
**Laparoscopic single anastomosis duodenal-ileal bypass with sleeve (SADI-S)**	CPT-4: * 43999
**Biliopancreatic Diversion with Duodenal Switch (BPD/DS) or Gastric Reduction Duodenal Switch (BPD/GRDS)**	CPT-4: 43845, (without Duodenal Switch)<br>ICD-9-CM: 45.91, 45.51, 43.89<br>ICD-10: 0D190Z9, 0DB60ZZ, 0DB80ZZ
**Open Vertical-banded gastroplasty (VBG)**	CPT: 43842 "Vertical-banded gastroplasty, not performed anymore", VGB procedures are essentially no longer performed.<br>ICD-9-CM: 44.68<br>ICD-10: 0DQ64ZZ
**Obesity**	ICD-9-CM: 278.0x, 278.01, V77.8, V85.41, V85.42, V85.43, V85.44, V85.45,<br>ICD-10: E66.xx, Z68.4x
**Morbid obesity**	ICD-9: 278.01<br>ICD-10: E66.01

CPT-4, Current Procedure Terminology (American Medical Association, Chicago, IL),
ICD-9-CM, International Classification of Diseases, Ninth Revision, Clinical Modification,

ICD-10-CM, International Classification of Diseases, Tenth Revision, Clinical Modification.
* Currently, there is no specific CPT code that describes the SADI bariatric surgery procedure. However, some surgeons may use an unlisted CPT code such as 43999 (Unlisted procedure, digestive system) to report the procedure.

Table S3. Procedure Codes Used to Identify Open and Laparoscopic Revisional Bariatric Surgery.

Description	Code	Code Type
**Laparoscopy, surgical, gastric restrictive procedure; removal of adjustable gastric restrictive device component only**	43772	CPT-4
**Revision of gastrojejunal anastomosis (gastrojejunostomy) with reconstruction, with or without partial gastrectomy or intestine resection; with vagotomy**	43865	CPT-4
**Revision of gastroduodenal anastomosis (gastroduodenostomy) with reconstruction; without vagotomy**	43850	CPT-4
**Revision of gastroduodenal anastomosis (gastroduodenostomy) with reconstruction; with vagotomy**	43855	CPT-4
**Laparoscopy, surgical, gastric restrictive procedure; revision of adjustable gastric restrictive device component only**	43771	CPT-4
**Laparoscopy, surgical, gastric restrictive procedure; removal and replacement of adjustable gastric restrictive device component only**	43773	CPT-4
**Revision of gastrojejunal anastomosis (gastrojejunostomy) with reconstruction, with or without partial gastrectomy or intestine resection; without vagotomy**	43860	CPT-4
**Revision, open, of gastric restrictive procedure for morbid obesity, other than adjustable gastric restrictive device (separate procedure)**	43848	CPT-4
**Laparoscopy, surgical, gastric restrictive procedure; removal of adjustable gastric restrictive device and subcutaneous port components**	43774	CPT-4
**Gastric restrictive procedure, open; removal of subcutaneous port component only**	43887	CPT-4
**Gastric restrictive procedure, open; revision of subcutaneous port component only**	43886	CPT-4
**Gastric restrictive procedure, open; removal and replacement of subcutaneous port component only**	43888	CPT-4
**Laparoscopic removal of gastric restrictive device(s)**	44.97	ICD-9
**Laparoscopic revision of gastric restrictive procedure**	44.96	ICD-9
**Open revision of RYGB**	44.5	ICD-9
**Gastric bypass procedures revision**	0D160*/	ICD-1
